# Efficacy of atosiban for repeated implantation failure in frozen embryo transfer cycles

**DOI:** 10.1038/s41598-023-36286-y

**Published:** 2023-06-07

**Authors:** Xiufang Li, Yanbo Du, Xu Han, Huidan Wang, Yan Sheng, Fang Lian, Qingfeng Lian

**Affiliations:** 1 Center for Reproductive Medicine, Shandong University, Jinan, 250021 China; 2 The First Clinical College, Shandong University of Traditional Chinese Medicine, Jinan, 250014 China; 3 Reproductive and Genetic Center of Integtated Traditional and Western Medicine, Affiliated Hospital of Shandong University of Traditional Chinese Medicine, Jinan, 250011 China; 4 Department of Radiology, Shandong Provincial Hospital Affiliated to Shandong First Medical University, Jinan, 250021 China

**Keywords:** Endocrinology, Medical research

## Abstract

Atosiban was commonly added to improve pregnancy outcomes of patients with repeated embryo implantation failure (RIF). In this study, we aimed to investigate the effect of atosiban before transferring the frozen-thawed embryo to RIF patients. This retrospective study was conducted in the Hospital for Reproductive Medicine affiliated to Shandong University from August 2017 to June 2021. A total of 1774 women with a history of RIF undergoing frozen embryo transfer (FET) were included in this study. All the participants were classified into atosiban or control group: Group A included 677 patients who were administered atosiban intravenously 30 min prior to FET with a dose of 37.5 mg; Group B included 1097 patients who received no atosiban before the transfer. There were no significant differences observed in the live birth rate (LBR) (39.73% vs. 39.02%, *P* = 0.928) between the two groups. Other secondary outcomes including biochemical pregnancy rate, clinical pregnancy rate, implantation rate, clinical miscarriage rate and preterm birth rate were similar between the two groups (all *P* > 0.05). However, subgroup analysis demonstrated significantly higher preterm birth rates in the control group compared with the atosiban group (0 versus 3.0%, *P* = 0.024) in the natural FET cycles. Atosiban may not improve pregnancy outcomes of RIF patients in FET cycles. However, the effects of Atosiban on pregnancy outcomes should be assessed in clinical trials with larger sample sizes.

## Introduction

In vitro fertilization (IVF) treatment is an effective treatment for various female infertility, and ovarian stimulation is used in most majority of IVF cycles so that multiple embryos are available for frozen embryo transfer (FET)^1^. However, the first imperative consideration for most top reproductive centers worldwide is to increase the clinical pregnancy rate and live birth rate of IVF. Recurrent implantation failure (RIF) results from repeated failed cycles of IVF or intracytoplasmic sperm injection (ICSI) treatment. RIF is only for patients undergoing assisted reproductive technology (ART). The precise definition of RIF remains controversial, but the most commonly described as “two or more failed cycles” or “three or more failed treatment cycles”^2^. Among patients undergoing IVF cycles, the incidence of RIF is about 15%^3^. It is estimated that 5% of patients suffer from recurrent pregnancy loss, of which 75% are diagnosed as RIF.The causes of RIF are complex, including poor embryo quality, advanced maternity age, uterine factors (such as endometrial polyps, intrauterine adhesions and submucosal fibroids), and hydrosalpinx^4,5^_,_ which may affect many molecular and physiological mechanisms involved in dynamic endometrial-blastocyst interactions^6^. Implantation is a multipart process that requires stability in the adhesion of the blastocysts, invasion of trophoblast cells, and immune regulation. A successful pregnancy strongly depends on the receptivity and structure of the endometrium. Due to the complexity of the implantation process, the evaluation of the causes of RIF should be analyzed on several levels. Although the exact pathogenesis of RIF is unclear, many studies have shown the angiogenesis and immunomodulatory factor disorders, the association between hormone imbalance, specific genetic polymorphisms, and the occurrence of RIF. Although many RIF women have undergone numerous clinical examinations and tried every possible treatment, they are still unable to conceive. In recent years, the incidence rate of RIF in infertility patients has continued to rise, which has become a new major challenge 8. RIF also brings a heavy financial burden and deeply impacts patients' physical and mental health. RIF is a serious challenge for both patients and fertility specialists in the field of ART.

Intrauterine environment and embryo quality are the two main factors that affect the embryo implantation^7^. However, RIF often occurs even in patients with high-quality embryos; the potential problems in endometrial receptivity must be emphasized when general pathology and uterine pathology have been ruled out. The ideal uterine conditions for embryo implantation require moderate uterine contractions and adequate blood supply to endometrium^8,9^. Excessive uterine contractions may reduce the implantation rate in ART cycles, and even expel the embryo to be expelled from the uterine cavity^10^. Zhu et al.^11^ reported that increased frequency of uterine peristaltic waves pushes mock embryos from the uterine cavity to the oviducts. Several recent studies have demonstrated that the frequency of endometrial peristalsis is increased in the frozen-thawed embryo transfer cycle of RIF women^12,13^. Therefore, inhibition of uterine contractions is predicted to effectively improve pregnancy outcomes in RIF patients.

Atosiban, a vasopressin V1A and a mixed oxytocin receptor antagonist, has been registered for the treatment of imminent premature birth with minimal side effects^14^. It has a similar structure to the oxytocin receptor. V1aR competitively binds to oxytocin receptors on the membrane of uterine leiomyoma, embryolemma and deciduous cells, thereby inhibiting calcium (Ca^2+^) release from the sarcoplasmic reticulum and prohibiting calcium entry. Oxytocin can trigger the production of prostaglandin F2 alpha (PGF2α) which has a paracrine action on the uterus, leading to contractions. Atosiban combined oxytocin vasopressor antagonism function reduces uterine contractility with simultaneous reduction in prostaglandin F2 alpha (PGF2α) production and improved endo-myometrial perfusion. Concerning atosiban safety, Lamon et al.^15^ discovered it is the safest tocolytic for Maternal and Fetal safety compared to other tocolytics. Yu et al.^16^ compared the safety of atosiban with conventional treatment (including indomethacin, β-agonists, magnesium sulfate, and calcium channel blockers, alone or in combination) of the threatened preterm labor, and the result showed no maternal adverse events in the atosiban treatment group. However, maternal treatment-related adverse events were observed, including cardiac arrhythmias, central nervous system disorders, and one case of fetal tachycardia with increased heart rate in conventional treatment groups. Recently published studies^17^ proved atosiban had no adverse effect on human spermatozoa and endocrine and also had a good embryonic safety profile in IVF cycles. Pierzynski et al.^18^ first reported that atosiban was administered to a 42-year-old female before embryo transfer and resulted in successful pregnancy, who had previously undergone 8 failed transplants of 12 high-quality embryos. Further studies reported atosiban as a promoter of endometrial receptivity, which increased the implantation rate and clinical pregnancy rate resulting in a desirable pregnancy outcome in patients with RIF during FET cycles^13,19,20^. A prospective cohort study showed that atosiban could reduce the number of uterine contractions and then increase the implantation and pregnancy rates in patients with RIF undergoing IVF/embryo transfer^13^. A Randomized Clinical Trial (RCT) study involving 194 infertile women by Tang et al.^9^ found that atosiban treatment before fresh embryo transfer might not improve the live birth rate in RIF patients.

Therefore, whether the utilization of atosiban contraction increases the pregnancy and implantation rates in FET or not remains controversial. In this study, we conducted a retrospective analysis on 1774 RIF patients in FET cycles to study the effects of atosiban on pregnancy outcomes.

## Materials and methods

This retrospective study analyzed records of 1774 FET cycles admitted to the Hospital for Reproductive Medicine affiliated with Shandong University from May 2017 to June 2021. The recruited patients were divided into two groups: the atosiban group (n = 677) and the control group (n = 1097), according to whether administered atosiban or not before FET. The experimental procedures were conducted in strict accordance with the Helsinki Declaration. Ethical approval was sought from the Affiliated Reproductive Hospital of Shandong University. All participants in this study informed of written consent. Inclusion criteria were as follows: (1) ≥ 2 implantation failures; (2)  ≥ 1good-quality blastocyst; (3) no difficulties in FET. Exclusion criteria were as followed: (1) with the history of uterine anomaly including uterine malformation, intrauterine adhesions, and endometrial polyps; (2) chromosomal anomalies and recurrent spontaneous abortion; (3) hydrosalpinx; (4) endocrine disorders such as hyperthyroidism, hypothyroidism, and hyperprolactinemia.

### Procedures

The participants were further divided into subgroups according to the regimens for endometrium preparation: natural cycle, hormone replacement therapy (HRT) cycle, suppression HRT cycle, and ovulation induction cycle. The natural cycle was only feasible for women with spontaneous ovulatory cycles. Cycle monitoring required several pelvic ultrasound scans to confirm the follicular development and time the commencement of urine testing to detect the luteinizing hormone (LH) surge. FET was performed 5 days after ovulation.

HRT cycle (suppression HRT) in which the endometrium was artificially prepared using sequentially administered exogenous estrogen and progesterone. In patients who maintained ovarian function, a gonadotrophin‐releasing hormone agonist (GnRHa) was used to temporarily suppress the ovarian function and render the patients functionally agonadal prior to inducing artificial cycles with estrogen and progesterone. With this method, oral exogenous estrogen administered on day 3 of the menstrual cycle prevented follicular recruitment by suppressing follicle-stimulating hormone (FSH) and avoiding spontaneous ovulation. The initial dose of estradiol valerate was 4 mg daily for 5 days, then 6 mg for the following 5 days, and 8 mg maximally, which was modulated according to the endometrial thickness and the serum levels of estradiol (E2). Dydrogesterone 40 mg/day (Abbott, the Netherlands) and progesterone capsule 200 mg/day (Laboratoires Besins International, France) as luteal phase support were added when the endometrial thickness reached 6.5 mm or more, and FET was carried out 5 days later. If pregnant, estrogen and progesterone must be continued until the placenta is established to replace the absent corpus luteum.The ovulation induction cycle used human menopausal gonadotrophins (HMG) or Letrozol tablets to induce ovulation and prepare the endometrium,and embryo transfer was conducted on the 5th day after ovulation. This approach could be offered to patients with irregular or anovulatory cycles undergoing FET.

The patients in the atosiban group were intravenously administered with atosiban (7.5 mg/mL; 5 mL of the agent was dissolved in 100 mL normal saline; Ferring Pharmaceuticals, Switzerland) 30 min prior to FET and the infusion was continued after FET with an infusion rate of 18 mg/h for 1–2 h. The total administered dose was37.5 mg. No atosiban was administered to the patients in the control group before FET.

### Outcome measures

The primary outcome was live birth rate, and the secondary outcomes included biochemical pregnancy, clinical pregnancy, implantation, miscarriage, ectopic pregnancy, multiple pregnancy and preterm birth rates. Live birth was defined as the delivery of any viable infant at 28 weeks or more of gestation after embryo transfer. Biochemical pregnancy was diagnosed when the serum hCG reached 25 IU/L at 12 days after FET. Clinical pregnancy was defined as the presence of at least one gestational sac on ultrasound About 33 days after the transplant. The miscarriage rate was defined as the number of miscarriages before 28 weeks divided by the number of women with clinical pregnancy. The preterm birth rate was defined as the percentage of birth before 34 weeks in women with live birth. All pregnant women were followed up for the pregnancy outcome after miscarriage or delivery.

### Blastocyst morphology evaluation

Blastocyst morphology was evaluated based on the Gardner grading system, which was divided into 6 periods according to blastocoel formation (Supplemental Table S1)^21^. Blastocysts with scores ≥ stage 4 (4BC) and blastocysts with score ≥ stage 4 (4BC) are defined as high-quality blastocysts.

### Statistical analysis

In data analysis, categorical data were presented as frequency and percentage, and the between-group differences were assessed using the chi-square test or Fisher’s exact test as appropriate. Continuous data were expressed as means ± standard deviation (SD) for normally distributed variables and were analyzed using t-test. Continuous data were expressed as median and interquartile range for non-normally distributed variables and were analyzed using Mann–Whitney U test. Data analyses were done using SPSS 22.0 (IBM, Armonk, NY, USA). *P* < 0.05 were considered as statistically significant.

### Ethics approval

The study protocol was approved by the Ethics Committee of the Reproductive Hospital Affiliated to Shandong University and adhered to relevant ethical guidelines. The studies involving human participants were reviewed and approved by the research ethics committee of Reproductive Hospital affiliated to Shandong University. The patients/participants provided their written informed consent to participate in this study.

## Results

A total of 1774 FET cycles with RIF were included in this study were available during the study period. The baseline characteristics of the patients are presented in Table 1. There were no significant differences in body mass index (BMI), duration of infertility, and baseline hormone values, including E2 and AMH (24.05 ± 3.42 vs. 23.47 ± 3.31, *P* = 0.575; 4.04 ± 3.11 vs.4.03 ± 2.74, *P* = 0.095; 33.66 ± 11.75 pg/ml vs. 34.90 ± 11.97 pg/ml,* P* = 0.064; 3.61 ± 2.30 ng/mL vs. 4.14 ± 2.25 ng/mL,* P* = 0.868) between two groups. The endometrial thickness, E2 on Luteal conversion day, and the number of embryos transferred cycle were comparable between the two groups (0.94 ± 0.15 cm vs. 0.93 ± 0.16 cm, *P* = 0.254; 226.70 ± 150.80 pg/ml vs. 227.03 ± 151.14 pg/ml, *P* = 0.622; 2.84 ± 1.11 vs. 2.84 ± 0.86, *P* = 0.942). The percentages of primary and secondary infertility, IVF and ICSI, and D5 blastocyst and D6 blastocyst were also comparable between the two groups (41.36% vs. 44.30%, 58.64% vs. 55.70%, *P* = 0.236; 64.40% vs. 66.00%, 35.60% vs. 34.00%, *P* = 0.514; 60.86% vs. 62.44%, 39.14% vs. 37.56%, *P* = 0.513). Patients in group A are older than group B (32.86 ± 4.79 vs. 31.69 ± 4.63, *P* = 0.012). There were significant differences in basal endocrine FSH and LH between the two groups (6.94 ± 1.66 IU/L vs. 6.48 ± 1.46 IU/L, *P* <0.001; 5.18 ± 2.37 IU/L vs. 5.70 ± 2.63 IU/L, *P* <0.001). The number of transferred embryos in group A was significantly less than in group B (89.51% vs. 85.60%, 10.49% vs. 14.40%, *P* = 0.02).

**Table 1:** Comparison of baseline data of the two groups.

	GROUP A (n = 677) Mean (SD)	GROUP B (n = 1097) Mean (SD)	Two-tailed *P* value
Age (SD) (year)	32.86 (4.79)	31.69 (4.63)	0.012
BMI (SD)	24.05 (3.42)	23.47 (3.31)	0.575
Duration of infertility (year)	4.04 (3.11)	4.03 (2.74)	0.095
Laboratory tests			
FSH (SD) (IU/L)	6.94 (1.66)	6.48 (1.46)	< 0.001
LH (SD) (IU/L)	5.18 (2.37)	5.70 (2.63)	< 0.001
E2 (SD) (Pg/ml)	33.66 (11.75)	34.90 (11.97)	0.064
AMH (SD) (ng/ml)	3.61 (2.30)	4.14 (2.25)	0.868
Infertility			
Primary infertility—no./total no. (%)	280/677 (41.36%)	486/1097 (44.30%)	0.236
Secondary infertility—no./total no. (%)	397/677 (58.64%)	611/1097 (55.70%)	
Fertilization			
IVF—no./total no. (%)	436/677 (64.40%)	724/1097 (66.00%)	0.514
ICSI—no./total no. (%)	241/677 (35.60%)	373/1097 (34.00%)	
Endometrial thickness(cm)	0.94 (0.15)	0.93 (0.16)	0.254
E2 on Luteal conversion day(SD) (pg/ml)	226.70 (150.80)	227.03 (151.14)	0.622
Stage of embryo transferred—no./total no. (%)			
D5 blastocyst	412/677 (60.86%)	685/1097 (62.44%)	0.513
D6 blastocyst	265/677 (39.14%)	412/1097 (37.56%)	
No. of embryos transferred cycle(SD)	2.84 (1.11)	2.84 (0.86)	0.942
No. of embryos transferred			
One embryo—no./total no. (%)	606/677 (89.51%)	939/1097 (85.60%)	0.02
Two embryos—no./total no. (%)	71/677 (10.49%)	158/1097 (14.40%)	

Pregnancy outcomes were similar in groups A and B after multivariate analysis, as shown in Table 2. The LBRs were similar (39.73% vs. 39.02%, *P* = 0.928), even after adjustment for confounding factors (adjusted odds ratio [aOR] 1.010, 95% confidence interval [CI]0.807–1.266). Clinical pregnancy rates were not significantly different (51.26% vs. 49.86%, *P* = 0.881) after adjusting for confounding factors (aOR 0.984, 95%CI 0.791–1.223). Biochemical pregnancy rates (61.15% vs. 59.71%, *P* = 0.542), Implantation rate (49.20% vs. 46.45%, *P* = 0.248), miscarriage rates (22.48% vs. 19.56%, *P* = 0.994), ectopic pregnancy rates (0.3% vs. 0.5%, *P* = 0.695), and preterm birth rate (2.95% vs. 4.19%, *P* = 0.288) were also similar.

**Table 2:** Comparison of clinical pregnancy outcomes between the two groups.

	GROUP A (n = 677) Mean (SD)	GROUP B (n = 1097) Mean (SD)	Croud OR^a^ (95% CI^b^)	*P* value	Adjusted OR^c^ (95% CI)	*P* value
Clinical outcome (%)						
Biochemical pregnancy rate	414/677 (61.15)	655/1097 (59.71)	1.062 (0.873–1.292)	0.549	0.933 (0.7472–1.166)	0.542
Clinical pregnancy rate	347/677 (51.26)	547/1097 (49.86)	1.057 (0.873–1.281)	0.591	0.984 (0.791–1.223)	0.881
Implantation rate	368/748 (49.20)	583/1255 (46.45)	1.116 (0.931–1.338)	0.248	–	–
Clinical miscarriage rate	78/347 (22.48)	107/547 (19.56)	1.192 (0.858–1.657)	0.310	1.001 (0.691–1.451)	0.994
Ectopic pregnancy rate	1/347 (0.3)	3/547 (0.5)	0.524 (0.054–5.059)	1.000	0.565 (0.033–9.825)	0.695
Preterm birth rate	20/677 (2.95)	46/1097 (4.19)	0.696 (0.408–1.186)	0.198	1.403 (0.751–2.623)	0.288
Live birth rate	269/677 (39.73)	428/1097 (39.02)	1.031 (0.847–1.254)	0.764	1.010 (0.807–1.266)	0.928

^a^OR, odds ratio.

^b^CI, confifidence interval.

^c^Adjusted by age, FSH, LH, and number of embryos transferred in FET.

As shown in Tables 3 and 4, subgroup analyses were performed to compare the effect of atosiban among the different endometrium preparation regimens and causes of infertility. There were no significant differences in live birth rate, clinical pregnancy, clinical miscarriage rate, and ectopic pregnancy between the atosiban and the control groups in subgroups(all *P* > 0.05). The subgroup analysis revealed significantly lower preterm birth rates in the atosiban group in natural FET cycles (Table 3) (0 versus 3.0%, respectively, *P* = 0.024). However, the sample size was insufficient to show significant differences between the subgroups according to the treatment group.

**Table 3:** Effect of atosiban in different endometrium preparation regimens on pregnancy outcomes.

Variable	Natural cycle			HRT cycle			Suppression HRT cycle			Ovarion induction cycle		
	Group A (n = 187)	Group B (n = 270)	*P* value	Group A (n = 194)	Group B (n = 322)	*P* value	Group A (n = 168)	Group B (n = 299)	*P* value	Group A (n = 128)	Group B (n = 206)	*P* value
Clinical pregnancy rate	99/187 (52.9%)	133/270 (49.3%)	0.448	102/194 (52.6%)	171/322 (53.1%)	0.758	78/168 (46.4%)	154/299 (51.5%)	0.335	68/128 (53.1%)	89/206 (43.2%)	0.091
Clinical miscarriage rate	17/99 (17.2%)	20/133 (15.0%)	0.718	23/102 (22.5%)	41/171 (24.0%)	0.883	19/78 (24.4%)	29/154 (18.8%)	0.391	19/68 (27.9%)	17/89 (19.1%)	0.250
Ectopic pregnancy rate	1/99 (1.0%)	0	0.268	0	1/171 (0.6%)	1	0	1/154 (0.6%)	1	0	1/89 (1.1%)	1
Preterm birth rate	0	8/270 (3.0%)	0.024	11/194 (5.7%)	18/322 (5.6%)	1	7/168 (4.2%)	11/299 (3.7%)	0.806	2/128 (1.6%)	9/206 (4.4%)	0.215
Live birth rate	81/187 (43.3%)	112/270 (41.5%)	0.701	80/194 (41.2%)	128/322 (39.8%)	0.781	59/168 (35.1%)	120/299 (40.1%)	0.322	49/128 (38.3%)	68/206 (33.0%)	0.347

**Table 4:** Effect of atosiban in different cause of infertility on pregnancy outcomes.

Variable	Tubal factor			Endometriosis			Male factor			Other		
	Group A (n = 520)	Group B (n = 769)	*P* value	Group A (n = 45)	Group B (n = 88)	*P* value	Group A (n = 53)	Group B (n = 125)	*P* value	Group A (n = 59)	Group B (n = 115)	*P* value
Clinical pregnancy rate	267/520 (51.3%)	371/769 (48.2%)	0.281	26/45 (57.8%)	43/88 (48.9%)	0.363	24/53 (45.3%)	71/125 (56.8%)	0.190	30/59 (50.8%)	62/115 (53.9%)	0.750
Clinical miscarriage rate	58/267 (21.7%)	65/371 (17.5%)	0.188	6/26 (23.1%)	11/43 (25.6%)	1	5/724 (20.8%)	19/71 (26.8%)	0.786	9/30 (30.0%)	12/62 (19.4%)	0.294
Ectopic pregnancy rate	1/267 (0.4%)	3/371 (0.3%)	0.644	0	0	1	0	0	1	0	0	1
Preterm birth rate	16/520 (3.1%)	27/769 (3.5%)	0.753	1/45 (2.2%)	6/88 (6.8%)	0.422	2/53 (3.8%)	8/125 (6.4%)	0.725	1/59 (1.7%)	5/115 (4.3%)	0.665
Live birth rate	209/520 (40.2%)	301/769 (39.1%)	0.728	20/45 (44.4%)	31/88 (35.2%)	0.348	19/53 (35.8%)	51/125 (40.8%)	0.616	21/59 (35.6%)	45/115 (39.1%)	0.742

## Discussion

RIF is a challenging condition in the field of IVF^22^. Methods to improve endometrial receptivity include adjuvant drug therapy (aspirin, low molecular weight heparin, vitamin E, and antibiotics), hormonal drug therapy (estrogen and progesterone), Traditional Chinese medicine(TCM), surgical treatment (laparoscopy and hysteroscopy), immunotherapy (passive immunity, active immunity, prednisone, and granulocyte colony-stimulating factor), and psychological treatment^23,24^. These methods were effective for some RIF patients; However, the efficacy of these treatments remains controversial. The present retrospective study analyzed the effects of atosiban on the pregnancy outcomes of patients with RIF in FET cycles. A total of 1774 patient cases were included in this study. The clinical data between the two groups were analyzed. The pregnancy outcomes were similar without significant differences after adjustment for confounding factors (all *P* > 0.05). The result is in agreement with previous studies, which means atosiban was no benefit to ART outcomes. Kim et al.^25^ performed a meta-analysis, and they suggested that the administration of atosiban on the day of ET was ineffective on the clinical pregnancy rate and abortion rate, although it could improve the implantation rate. Meanwhile, another two meta-analyses recently revealed that atosiban could increase the clinical pregnancy and embryo implantation rates of IVF (FET) in patients with RIF and play a limited role to the general population^26,27^. However, these studies did not detect the endometrial peristalsis during medication. Therefore, the inappropriate design of research methods and inadequate analysis reduced the reliability of these conclusions.

Uterine peristalsis or contraction plays a vital role in the human reproductive process. The success of embryo transfer may be affected by uterine contractions, endometrial receptivity, and embryo quality^10^. Fanchin et al.^8^ discovered a stepwise decrease in pregnancy rates and implantation rates when the uterine contractions frequency increased from 3 to > 5 per minute. Patients with fewer uterine contractions before embryo transfer have a higher IVF success rate, and the pregnancy rate of patients whose uterine contractions of < 3/min before embryo transfer was significantly higher^28^. However,the patients with uterine contractions ˃3 times per minute accounted for 6.2% (18 out of 292) of pregnancy losses^28^. In the present study, no significant differences were found in the outcomes of FET between the two groups. Oxytocin plasma concentrations increase during the follicular phase, reaching the maximum around the time of ovulation, and subsequently decrease during the luteal phase^29,30^. Oxytocin of endometrial origin was thought to stimulate myometrial activity and involve in sperm and egg transport and implantation. Atosiban, an oxytocin antagonist, can decrease the amplitude and frequency of uterine contractions and promote implantation by preventing early embryo expulsion^31^. Wu et al. discovered that atosiban was not significantly associated with pregnancy outcomes among all study infertile women (including one, two, or more embryo transfer cycles). For RIF women, the clinical and ongoing pregnancies were significantly increased with atosiban^32^. In 2011, chou et al. reported that a lower dosage of atosiban improved pregnancy outcomes of patients with RIF, but the number of recruited patients was small^20^. Another study by Wang et al.^33^ discovered RIF patients presented with higher clinical pregnancy rates with the administration of atosiban in IVF-ET cycles, without significant changes in ectopic rate, multiple-gestation pregnancy rate, and miscarriage rate. Ng et al.^34^ found that 800 patients receiving IVF were selected from Guangzhou, Hong Kong, and Ho Chi Minh City and admitted in a multicenter, randomized, and double-blind trial. They were divided into the atosiban group (n = 400) and the control group (n = 400) by a computerized random number generator. However, there were no significant differences inpregnancy outcomes. Another RCT study revealed that atosiban use before fresh embryo transfer could not improve the live birth rate in RIF patients^9^. These results were similar to our study. We didn’t observe any benefit of atosiban with RIF patients in FET cycles, which could be attributed to the short plasma half-life of atosiban injection, resulting in a limited therapeutic time window. Thus, suppression of uterine contractions did not persist after the injection. Although the preterm birth rate in the subgroup was significantly lower with atosiban in natural FET cycles, this may be related to the small sample size and sample deviation.

The level of prostaglandins in the plasma of patients with RIF increases, which can induce oxytocin secretion to lead to uterine contraction, and the times of embryo transfers was positively correlated with the frequency of uterine contractions^12^. Embryo transfer is the final step of FET, which may increase the local oxytocin and prostaglandin release^35^. The elevated frequency of uterine contractions in FET cycles is mainly due to prolonged procedures within the uterine cavity during the embryo transfer and on the day of FET, which may stimulate uterine contraction and result in a lower implantation rate and pregnancy rate. Difficulties in embryo transfer could affect the success rate of FET. In addition, the medical specialist performing the embryo transfer can inevitably touch the vaginal walls and the cervix during the transfer, which prompts the generation of oxytocins and PGF2a release. This will induce uterine contractions and push the embryos out of the uterine cavity^36^. Our results showed that, though the pregnancy rate in every subgroup of the atosiban group seemed to be higher, there was no significant difference compared to the control group.

The present clinical control study has limitations. Because of the non-prospective method without randomization and small sample size in subgroups, our evidence is not as strong as that of randomized controlled trials. Therefore, a randomized, prospective, placebo-controlled trial with a large sample size is needed to verify the present results and provide stronger evidence for further studies. Additionally, There was no measurement of uterine contractions and incomplete follow-up of congenital abnormalities of newborns in this study. The long-term efficacy and safety of atosiban are unknown.

In conclusion, our study suggested that treatment using atosiban prior to FET was not associated with significant beneficial outcomes for improving the LBR of RIF patients in FET cycles. The clinical application value and safety of atosiban need further study. Well-conducted RCTs focusing on RIF women are warranted.

## Supplementary Information

Supplementary Table S1.

## Data Availability

Data and materials are available. The datasets generated and/or analyzed during the current study are available from the corresponding author Qingfeng Lian upon request.

## Code availability

Sofware application was available.
