# Supplementary material for: Efficacy of atosiban for repeated implantation failure in frozen embryo transfer cycles
Source: Sci Rep. 2023 Jun 7;13:9277. doi: 10.1038/s41598-023-36286-y (PMC10247710; doi:10.1038/s41598-023-36286-y)
Supplement: Supplementary file 1 — Supplementary Table S1. [file 41598_2023_36286_MOESM1_ESM.docx]

**Supplemental Table 1 Blastocyst Morphology Evaluation**

|  | Grade | Description |
| --- | --- | --- |
| Developmental  Stage | Stage 1 | Early cavitary blastocyst with blastocyst cavity less than 1/2 of the embryo volume |
|  | Stage 2 | blastocyst cavity coelom greater than or equal to 1/2 of the volume |
|  | Stage 3 | fully expanded blastocyst, blastocyst cavity occupies the embryo |
|  | Stage 4 | expanded blastocyst, blastocyst cavity volume greater than an early embryo, zona pellucida thinning |
|  | Stage 5 | blastocyst being hatched, trophoblast begins to break through the zona pellucida |
|  | Stage 6 | hatched blastocyst, the blastocyst is completely hatched from the zona pellucida |
| Inner cell mass (ICM) | A | the inner cell mass is tight and the number of cells is large |
|  | B | the inner cell mass is loose and the number is small |
|  | C | the number of the inner cell mass is very small |
| Trophectoderm  (TE) | A | the number of cells is large and a tightly arranged cell layer is formed |
|  | B | the number of cells is small and the arrangement is loose |
|  | C | the trophectoderm is composed of sparse cells |
